# Day Hospital Mentalization-Based Treatment (MBT-DH) versus treatment as usual in the treatment of severe borderline personality disorder: protocol of a randomized controlled trial

**DOI:** 10.1186/1471-244X-14-149

**Published:** 2014-05-22

**Authors:** Elisabeth MP Laurenssen, Dieuwertje Westra, Martijn J Kikkert, Marc J Noom, Hester V Eeren, Anna J van Broekhuyzen, Jaap Peen, Patrick Luyten, Jan JV Busschbach, Jack JM Dekker

**Affiliations:** 1 Viersprong Institute for Studies on Personality Disorders (VISPD), Halsteren, The Netherlands; 2 Arkin, Amsterdam, The Netherlands; 3 De Viersprong, Amsterdam, The Netherlands; 4 NPI, Institute for Personality Disorders, Amsterdam, The Netherlands; 5 Department of Psychiatry, section Medical Psychology and Psychotherapy, ErasmusMC, Rotterdam, The Netherlands; 6 Faculty of Psychology and Educational Sciences, University of Leuven, Leuven, Belgium; 7 Research Department of Clinical, Educational and Health Psychology, University College London, London, UK; 8 Department of Clinical Psychology, VU University Amsterdam, Amsterdam, The Netherlands

**Keywords:** Mentalization-based treatment, Treatment as usual, Randomized controlled trial, Multi-site, Cost-effectiveness

## Abstract

**Background:**

Severe borderline personality disorder is associated with a very high psychosocial and economic burden. Current treatment guidelines suggest that several manualized treatments, including day hospital Mentalization-Based Treatment (MBT-DH), are effective in these patients. However, only two randomized controlled trials have compared manualized MBT-DH with treatment as usual. Given the relative paucity of data supporting the efficacy and cost-effectiveness of MBT-DH, the possible influence of researcher allegiance in one of the trials, and potential problems with the generalization of findings to mental health systems in other countries, this multi-site randomized trial aims to investigate the efficacy and cost-effectiveness of manualized MBT-DH compared to manualized specialist treatment as usual in The Netherlands.

**Methods/design:**

The trial is being conducted at two sites in The Netherlands. Patients with a DSM-IV-TR diagnosis of borderline personality disorder and a score of ≥ 20 on the Borderline Personality Disorder Severity Index were randomly allocated to MBT-DH or treatment as usual. The MBT-DH program consists of a maximum of 18 months’ intensive treatment, followed by a maximum of 18 months of maintenance therapy. Specialist treatment as usual is provided by the City Crisis Service in Amsterdam, a service that specializes in treating patients with personality disorders, offering manualized, non-MBT interventions including family interventions, Linehan training, social skills training, and pharmacotherapy, without a maximum time limit. Patients are assessed at baseline and subsequently every 6 months up to 36 months after the start of treatment. The primary outcome measure is the frequency and severity of manifestations of borderline personality disorder as assessed by the Borderline Personality Disorder Severity Index. Secondary outcome measures include parasuicidal behaviour, symptomatic distress, social and interpersonal functioning, personality functioning, attachment, capacity for mentalizing and quality of life. Cost-effectiveness is assessed in terms of the cost per quality-adjusted life year. Outcomes will be analyzed using multilevel analyses based on intention-to-treat principles.

**Discussion:**

Severe borderline personality disorder is a serious psychological disorder that is associated with high burden. This multi-site randomized trial will provide further data concerning the efficacy and cost-effectiveness of MBT-DH for these patients.

**Trial registration:**

NTR2175

## Background

Borderline Personality Disorder (BPD) is one of the most prevalent mental disorders [1]. In the general population 1 to 2% of adults are diagnosed with BPD. In psychiatric populations up to 10% of outpatients and 20% of inpatients are diagnosed with BPD [1,2]. BPD is also associated with high psychosocial and socio-economic costs [3,4]. The economic burden of disease associated with BPD is higher than that associated with depression, and comparable to that of patients with schizophrenia [1,4]. BPD is also associated with high psychiatric comorbidity, particularly depression, anxiety disorders, eating disorders, substance abuse [5-7] and various other personality disorders [5,6,8-13], often in combination with high levels of acting-out (e.g., suicidality) [14] and/or functional impairment [15-17]. The lifetime risk for completed suicide associated with BPD may be as high as 10% [14]. Together, these findings emphasize the need for the development of effective treatments for this severe disorder.

In recent years, a number of evidence-based specialist treatments for BPD have been developed and evaluated [1]. These include dialectical behaviour therapy [18], schema therapy [19], transference-focused psychotherapy [20,21], Systems Training for Emotional Predictability and Problem Solving (STEPPS) [22], and Mentalization-Based Treatment (MBT) [23]. Various reviews [1,24,25] and treatment guidelines [26-28] recommend these treatments for patients with BPD.

The present study focuses on MBT as developed by Bateman and Fonagy in the United Kingdom [23,29,30]. MBT is a promising psychodynamic treatment that is rooted in attachment and mentalizing approaches. Briefly, mentalizing refers to the capacity to interpret the self and others in terms of internal mental states such as feelings, emotions, wishes, desires, attitudes and values. This capacity is typically acquired in attachment relationships, and is associated with feelings of self-agency, affect regulation and resilience in the face of adversity. A growing body of research suggests that impairments in mentalizing can be seen as a core feature of BPD, as patients with BPD typically fail to make sense of their own internal experiences and those of others, particularly in contexts characterized by high levels of arousal. This results in emotional instability, impulsive behaviour, and the use of self-defeating strategies in an attempt to cope with these feelings (such as self-harm, substance abuse and promiscuity).

So far, two types of MBT have been empirically investigated: intensive outpatient MBT (MBT-IOP) [29] and day hospital MBT (MBT-DH). Both MBT-IOP and MBT-DH consist of a treatment phase and a maintenance phase, each lasting a maximum of 18 months. The treatment phase of MBT-DH, the focus of the current study, consists of a day hospital treatment (five days per week) that includes daily group psychotherapy, weekly individual psychotherapy, individual crisis management from a mentalizing perspective, art therapy twice a week, mentalizing cognitive therapy and writing therapy. The maintenance phase in MBT-DH consists of a one-day follow-up treatment program combined with intermittent individual follow-up appointments, with the frequency reduced over time (step down).

At the time the current study was designed (2007), there was only one randomized controlled trial (RCT) investigating the efficacy and cost-effectiveness of MBT-DH compared to treatment as usual (that is, standard psychiatric care) conducted by the developers of MBT [31]. This study randomized 38 BPD patients to either MBT-DH or TAU, which consisted of standard treatment offered in the UK in general psychiatric services and comprised (a) regular psychiatric review with a senior psychiatrist when necessary (on average twice a month); (b) inpatient admission when necessary, with discharge to non-psychoanalytic psychiatric partial hospitalization focusing on problem solving; followed by (c) outpatient and community follow-up as standard aftercare [31]. Results showed that MBT-DH was superior to TAU on all major outcome variables, that is, depressive symptoms, suicide attempts and self-harm, number of inpatient days, and social and interpersonal functioning. These results were maintained during the 18-month follow-up period [32]. Five years after discharge from MBT, the MBT-DH group still showed superiority over TAU on suicidality, diagnostic status, service use, use of medication, global functioning scores above 60 (on the Global Assessment of Functioning [GAF] Scale), and vocational status [33]. For example, 74% of the patients in the TAU condition had made at least one suicide attempt, in comparison with only 23% in the MBT-DH group. And at the end of the follow-up period, 13% of the MBT-DH patients met the diagnostic criteria for BPD, compared to 87% of the TAU group. Before treatment the total health related-costs for the MBT-DH group ($44,947) and the TAU group ($52,563) were comparable; after 18 months of treatment the costs were reduced to $27,303 in MBT-DH and $30,976 in TAU. During the 18-month follow-up, costs further diminished sharply. After 18 months follow-up, the total health-related costs in the MBT-DH group were one-fifth of that for patients in the TAU condition: $3,183 for MBT-DH compared to $15,490 for TAU [32].

Since this original trial, two other trials focusing on MBT-DH have been published. An RCT in Denmark investigated the efficacy of MBT-DH compared with a less intensive manualized supportive group therapy combined with psycho-education and medication treatment in patients diagnosed with BPD [34]. In total, 58 patients were randomly allocated to MBT-DH and 27 patients to the specialist combined treatment. Results showed that both the intensive combined MBT treatment and the less-intensive supportive group therapy led to significant improvements on a variety of psychological and interpersonal measures, e.g., general functioning, depression, social functioning and number of diagnostic criteria met for BPD, with moderate to large effect sizes (*d* = 0.5 to 2.1). Contrary to the expectations of the researchers, however, MBT-DH was superior only on therapist-rated GAF [34]. No follow-up or cost-effectiveness data are yet available from this trial.

Additional evidence for the effectiveness of MBT-DH comes from a naturalistic study by Bales and colleagues [35] in The Netherlands. These authors investigated the effectiveness of 18-months of manualized MBT-DH in 45 patients with severe BPD and a high prevalence of comorbid Axis-I and Axis-II disorders. Results showed significant improvements in symptomatic distress, social and interpersonal functioning, and personality pathology and functioning; with moderate to large effect sizes (*d* = 0.7 to 1.7). These authors also showed that care consumption, defined as additional treatments and admissions during the last year before entry into and during MBT treatment, reduced significantly during and after treatment. Yet, the lack of a control group limits the possibility to draw conclusions from this study about the effectiveness of MBT-DH.

Hence, although there is some promising evidence supporting the efficacy and cost-effectiveness of MBT-DH, given the small number of studies, more research is urgently needed, particularly in light of the limitations of existing trials. First, one of the two RCTs was conducted by the developers of MBT [31], and thus researcher allegiance may have influenced this study. Second, it is unclear whether results from trials conducted in the UK and Denmark may generalize to The Netherlands, given the large differences in health care systems between these countries. For instance, standard psychiatric care may be more effective in The Netherlands than in the UK because of differences in the allocation of health care and the clinical training of health workers. For instance, standard care in the Netherlands includes more and thus more expensive evidence-based treatments compared to the UK, and there is more funding available per patient. This assumption leads to the expectation that differences between a specialist treatment such as MBT-DH and TAU may be smaller in The Netherlands, as they may be in Denmark, which may explain the lack of substantial differences between MBT and specialist standard care in the trial of Jørgensen et al [34]. This assumption is supported by recent findings that highly structured treatment programmes are associated with considerable effects in BPD patients, which are often comparable to the effects of specialist treatments such as MBT [33,34,36]. For instance, in a randomized trial of BPD patients, Bateman and Fonagy [29] found that MBT-IOP outperformed a manualized structured clinical management programme only at long-term follow-up in terms of effects on suicide attempts, severe incidents of self-harm, symptom severity, depression, interpersonal functioning and social adjustment. Hence, with regard to generalizability, a concern is the need for trials comparing MBT-DH to a credible TAU [29].

Further, the study by Jørgensen and colleagues [34] suffered from a number of important methodological limitations, such as a skewed randomization (with the majority of patients being randomized to MBT-DH) and the fact that the same therapists conducted treatments in both conditions (i.e., MBT-DH and supportive therapy), which may have led to “spill-over” effects.

Finally, none of the existing trials focused on the purported mechanism of change in MBT, that is, changes in attachment and mentalizing. Given the growing evidence for the role of common factors in psychotherapy, there is a pressing need to provide evidence for presumed mechanisms of change in current evidence-based treatments such as MBT [37].

### Research aims and hypotheses

The primary aim of the present study was to investigate the efficacy and cost-effectiveness of MBT-DH in comparison to a specialist TAU in The Netherlands. We expect both MBT-DH and specialist TAU to be effective on both primary and secondary outcomes. Yet, we expect MBT-DH to outperform TAU, particularly at 36 months follow-up. After 18 months of treatment we expect the costs in the MBT programme to be comparable to TAU; after 36 months we expect MBT-DH to outperform TAU (following Bateman & Fonagy [32]). Second, we will investigate purported mechanisms of change in MBT, focusing on changes in attachment and mentalizing. Results of this trial are expected to inform mental health professionals, patients, and policy makers.

## Methods/design

### Design

This study is a multi-site RCT comparing the efficacy and cost-effectiveness of manualized MBT-DH and a manualized specialist TAU in the treatment of BPD. The inclusion of patients started in March 2009 and ended in July 2012. The last follow-up measurement, at 36 months, will be in July 2015. Two mental health care centres agreed to participate in this study: Arkin en De Viersprong, both located in Amsterdam. Arkin is a large mental health care centre that specializes in the treatment of BPD. De Viersprong is a national institute for personality problems, offering specialized outpatient, day hospital, and inpatient psychotherapy for adolescent and adult patients with severe personality problems and disorders. Arkin agreed to run four MBT-DH groups and De Viersprong agreed to run two MBT-DH groups in the context of the trial, each consisting of nine patients. The City Crisis Service, which is part of Arkin, agreed to run the specialist TAU condition. The overall aim was to randomize 54 patients to MBT-DH and 54 patients to TAU.

### Ethics

A certified Medical Ethics Review Committee in The Netherlands has approved this study, registered under NL38571.078.12. This ethical approval covered all sites of data collection.

### Participants

Patients were referred for treatment by general practitioners, mental health care institutions, private practices and general hospitals. Inclusion criteria were (a) a BPD diagnosis as measured by the Structured Clinical Interview for DSM-IV Axis II Personality Disorders (SCID-II) [38] and (b) a total score on the Borderline Personality Disorder Severity Index (BPDSI) [39] of at least 20, reflecting severe BPD. Originally, a BPDSI score ≥ 25 was proposed as a cut-off point. However, it became clear quickly that this criterion was overly strict, as several patients who did not meet this criterion showed severe functional impairments. Hence, we decided to adopt a cut-off of 20. Exclusion criteria were kept to a minimum to ensure generalizability and to allow patients with severe BPD to be included. The exclusion criteria were: (a) the presence of an Axis-I disorder (as determined with the Structured Clinical Interview for DSM-IV Axis I Personality Disorders (SCID-I) [40]) that required specialist treatment, (b) organic brain disorder, (c) IQ below 80, and (d) inadequate mastery of the Dutch language.

### Procedure

**Figure 1:**
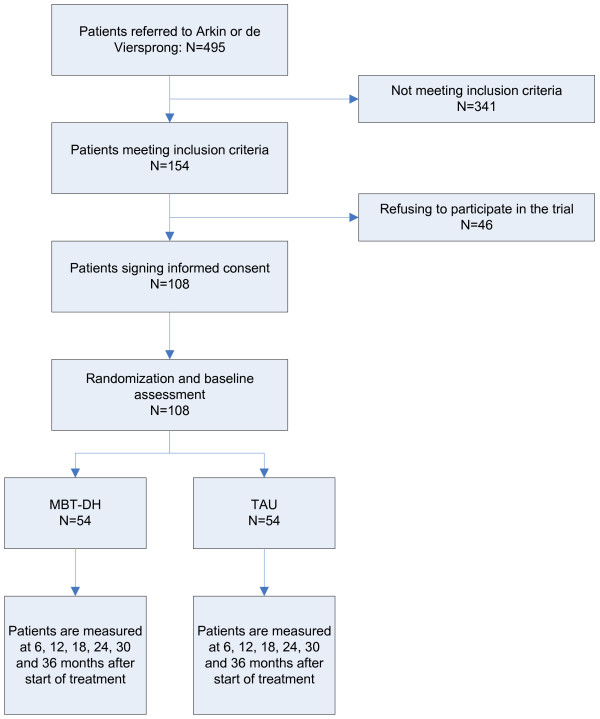
Expected patient enrolment in the study.

All patients referred to one of the mental health care institutes were invited to attend an intake appointment. Patients who were deemed eligible received more information about the study and, if they were interested, a new appointment was set during which the BPSDI [39], and SCID-II [38] interviews were administered and other eligibility criteria were assessed. If patients met inclusion criteria, they provided informed consent and were randomized to either MBT or TAU by an independent researcher using a computerized 1:1 algorithm. Patients were subsequently asked to complete the baseline measures (see Figure 1). It was preferred that patients completed the measures in the clinic; however if patients insisted, they were allowed to complete questionnaires at home, in which case patients were asked to return the questionnaires within a week by regular mail.

After baseline assessments, patients are assessed at 6, 12, 18, 24, 30 and 36 months after the start of treatment. At these time points, patients complete a battery of questionnaires and interviews. In addition, at 36 months, the SCID-I [40], SCID-II [38], BPDSI [39], the Substance use section of the Measurements in the Addictions for Triage and Evaluation (MATE) [41] and the Suicide and Self-Harm Inventory (SSHI) [23] are readministered by research assistants (MSc-level psychologists) who are blind to treatment condition. Patients are invited by telephone to come to the clinic to complete these assessments. If they do not respond or fail to attend the first appointment, up to three attempts are made to contact them by telephone to remind them. If patients refuse to complete the questionnaires at the clinic, they are allowed to complete them at home and return them by regular post; in this case, the SCID-I, SCID-II, BPDSI, MATE and SSHI can be completed over the telephone.

### Treatments

#### Day hospital MBT

MBT-DH is an intensive, manualized specialist treatment for patients with BPD that has been described in detail elsewhere [23,35]. Briefly, MBT-DH in this study consists of an intensive day-hospitalization programme of 18 months followed by a maximum of 18 months of maintenance mentalizing (group) therapy. The day-hospital program, covering five days per week and 4.5 hours per day, includes the following components: (a) implicit mentalizing groups (i.e., daily group psychotherapy and weekly individual psychotherapy, and individual crisis planning from a mentalizing perspective) and (b) explicit mentalizing groups (i.e., art therapy twice a week, mentalizing cognitive group therapy, and writing therapy). The week’s program is ended by a social hour and a community meeting. Patients can also consult a psychiatrist once a week and medication is offered following American Psychiatric Association guidelines.

The treatment goals of MBT-DH are: (a) to engage the patient in treatment; (b) to reduce psychiatric symptoms, particularly self-harm and parasuicidal behaviour; (c) to improve social and interpersonal functioning; and (d) to foster more appropriate health care usage and prevent hospital admissions and prolonged inpatient care. To achieve these goals, components of the programme focus on enhancing the capacity for mentalizing.

As outlined above, the assumption is that by enhancing their mentalizing capacity, particularly in high arousal contexts, patients will show improvements in understanding themselves and others, leading to a decreased need to rely on maladaptive coping strategies to deal with feelings of inner emptiness and “badness”, impulsivity and relationship conflicts.

The MBT teams consist of a total of three certified and registered psychologists, two psychotherapists, one psychiatrist, four sociotherapists, and a creative therapist. All clinicians who are involved in providing MBT-DH followed an MBT training programme led by a certified MBT trainer in The Netherlands, who also acted as a consultant before and during the study and provided supervision in the initial stages of the trial.

Adherence to the MBT treatment model is monitored in the following ways. First, in daily group reflections after the group therapy, therapists are stimulated to reflect on their adherence to the treatment model. Specifically, therapists are asked to reflect on whether their interventions have enhanced mentalizing, which interventions have not, and what alternative interventions might have been more successful. Second, every 6 months therapists complete, with regard to their own interventions and those of their co-therapist, the MBT Adherence and Competence Scale [42] for five consecutive days within one week after a group therapy session. Third, during team supervision (every 2 weeks), adherence to the treatment model is addressed using case material.

#### Treatment as usual

As noted, TAU is based on a manualized treatment for severe personality disordered patients [43]. When possible, patients in the TAU condition are treated by the City Crisis Service itself. When more specific treatment is needed (e.g., treatment for drug addiction or hospitalization), other treatment centres are sought as appropriate for patients’ specific needs. Additionally, if patients prefer to be treated in the area where they live, the intaker or crisis team aims to find an appropriate treatment for them in their own environment.

The City Crisis Service is specialized in providing emergency assistance and long-term aftercare treatment after crises and is composed of five psychiatrists, two psychologists, five mental health nurses and one system therapist. Treatment is mainly provided on an outpatient basis; day treatment or hospitalization is offered only when necessary. The team aims to deliver optimal care by matching the intensity of one or more interventions with the need of the patients. Interventions consist of supporting and structuring sessions, extensive diagnostic investigations, writing a crisis plan, family interventions, STEPPS [22] or Linehan training, social skills training or learning how to deal with aggression/impulse control, cognitive schema-focused or traditional insight-oriented treatment, pharmacotherapy, and inpatient treatment when deemed necessary. The outpatient team involved in the City Crisis Centre has a capacity of 50 patients. Because of the specialist nature of this service, the fact that its treatment was manualized, and its extensive experience with treating patients with BPD, we chose it as the provider of TAU for the present study. There is no maximum time limit for treatment. No adherence measures are available for TAU.

### Measurements

#### Demographic variables

At baseline, participants complete questions concerning their marital status, living situation, religion, education level, diploma, current job and working situation, and questions concerning the main earner in the family (relation to the patient, annual income, occupation, and source of income).

#### Primary outcome

The total score on the BPDSI [39] was chosen as the primary outcome measure. The BPDSI is a semi-structured interview, developed to assess BPD features as defined by DSM-III-R/DSM-IV criteria [44,45]. The purpose of the instrument is not to diagnose BPD, but to yield a quantitative index of the current severity of BPD features. The BPDSI contains the following nine subscales: (a) abandonment, (b) relationships, (c) identity, (d) impulsivity, (e) parasuicidal behaviour, (f) affective instability, (g) emptiness, (h) anger-control, and (i) dissociation and paranoid ideation. The BPDSI has been shown to be sensitive to change [39]. In a study of BPD patients, patients with other personality disorders, and patients with only Axis-I disorders, the BPDSI was highly reliable (intraclass correlation coefficient [ICC] = .93) and internally consistent (Cronbach’s α = .85).

#### Secondary outcomes

##### Symptomatic distress

Symptomatic distress is assessed with the SSHI, the Personality Assessment Inventory-Borderline (PAI-BOR), the Beck Depression Inventory (BDI-I), the Brief Symptom Inventory (BSI), the Outcome Questionnaire (OQ-45), and the MATE.

Suicide and self–harm are assessed with the SSHI [23]. The SSHI is a semi-structured interview assessing (a) the frequency and severity of suicidal acts in the past 6 months, and (b) the frequency and severity of acts of self-mutilation in the past 6 months. This interview poses specific questions not only about the number of acts but also about the dangerousness of these acts - that is, the presence or absence of another person at the time, the likelihood of being found, preparation for the act, and lethality of the act. Multiple acts over a short period of time – for example, frenzied self-cutting - are counted as a single act [31].

The Dutch version of the PAI-BOR [46,47] is part of the Personality Assessment Inventory [46] and consists of four subscales (each with six items), which reflect four characteristics of BPD: affective instability (AI), identity problems (IP), negative relationships (NR), and self-harm (SH). There are four response categories (0 = false, 1 = slightly true, 2 = mainly true, and 3 = very true). An example item is “Sometimes I feel very empty inside”. According to the manual of the PAI-BOR [46], a total PAI-BOR raw score of 38 or more indicates the presence of significant BPD features, whereas a score of 60 or more indicates typical borderline personality functioning. The internal consistency (Cronbach’s α) of the Dutch PAI-BOR is good (α = .81) and 6-month test-retest correlation is .78 [47].

The BDI-I is used to assess depressive symptoms [48]. The BDI-I is a self report instrument, which consists of 21 questions concerning depressive symptoms during the last week. Each question has a set of four possible answers, ranging in intensity from 0 to 3, for example, “I don’t feel sad” (0) to “I feel so sad or unhappy that I cannot bear it anymore” (3). The total scores are categorized: 0–9 no depression, 10–18 mild-moderate depression, 19–29 moderate-severe depression and 30–63 severe depression. The BDI-I has been shown to have high internal consistency (α > .80) is one of the most commonly used instruments to assess severity of depression [49].

General psychopathological symptoms are assessed with the Dutch version of the BSI [50,51]. The BSI is the short version of the Symptom Checklist-90. It consists of 53 items covering nine symptom dimensions (somatization, obsession-compulsion, interpersonal sensitivity, depression, anxiety, hostility, phobic anxiety, paranoid ideation, psychoticism) and yields three global indices of distress: Positive Symptom Distress Index, Positive Symptom Total, and Global Severity Index (GSI). Possible GSI scores range from 1 to 5, with higher scores indicating a higher level of psychological and emotional distress. Respondents have to rate each feeling item (e.g., “your feelings being easily hurt”) on a five-point scale ranging from 0 (not at all) to 4 (extremely), representing the intensity of distress during the past 7 days. Reliability of the Dutch version is good (Cronbach’s α ranging from .71 to .85) and the factor structure is comparable to that of the original versions of Derogatis [51].

The OQ-45 [52,53] was developed to capture three domains central to mental health: symptom distress, interpersonal relations and social role functioning. This self-report questionnaire consists of 45 items to be rated on a five-point Likert scale ranging from 0 (never) to 4 (almost always). Reliability and validity of this instrument have been demonstrated [52,53].

The substance use section of MATE [54] is used to assess substance abuse and dependency. This section, which is designed as an interview, asks about the use of psychoactive substances in the past month and during the lifetime. The interviewer in this study was an MSc-level psychologist. The inter-rater reliability ranges between 0.75 and 0.92 [55].

##### Social and interpersonal functioning

The Inventory of Interpersonal Problems (IIP-64) is used to assess interpersonal problems [56]. It is a self-report measure consisting of 64 items assessing eight dimensions of interpersonal problems: (1) domineering/controlling (e.g., “It is hard for me to take instructions from people who have authority over me”), (2) vindictive/self-centred (“It is hard for me to trust other people”), (3) cold/distant (“It is hard for me to show affection to others”), (4) socially inhibited (“It is hard for me to introduce myself to someone”), (5) non-assertive (“It is hard for me to be firm when I need to be”), (6) overly accommodating (“It is hard for me to be angry at others”), (7) self-sacrificing (“It is hard for me to be angry at someone I like”) and (8) intrusive/needy (“It is hard for me to be on my own”). Respondents are asked to consider each problem and to rate how distressing that problem has been on a scale ranging from 0 (not at all) to 4 (extremely). The IIP-64 possesses good psychometric properties, with Cronbach’s α of .96 for the total score and a test-retest reliability coefficient of .78 [57].

##### Personality functioning

Personality functioning is assessed with the Severity Indices of Personality Problems – Short Form (SIPP-SF) and the Dimensional Assessment of Personality Pathology – Short Form (DAPP-SF).

The SIPP-SF [58] is a dimensional self-report measure assessing the severity of personality pathology and has been developed for research purposes. The SIPP-SF aims to assess the core components of adaptive and maladaptive personality functioning. It consists of 60 items, which cluster into five higher-order domains: (1) self-control, (2) identity integration, (3) relational capacities, (4) social concordance and (5) responsibility [59]. The SIPP-SF asks respondents to think about the past 3 months and to answer the extent to which they agree with statements such as “I frequently say things I regret later”. Items are rated on a four-point Likert type scale ranging from 1 (fully disagree) to 4 (fully agree). High scores on the domains indicate better functioning. The SIPP-SF is a shortened version of the SIPP-118, which has good reliability (Cronbach’s α ranging from .62 to .89, M = .78) [59].

The DAPP-SF consists of 136 of the original 290 items of the DAPP-BQ [60,61]. The 136 items cover 18 personality disorder trait-based dimensions fitting into four broad higher order factors: emotional dysregulation, dissocial behaviour, inhibition and compulsivity [62]. The items are rated on a five-point Likert scale with scores ranging from 1 (very unlike me) to 5 (very like me). Both the DAPP-BQ and the DAPP-SF have good psychometric features [62].

##### Attachment and mentalizing

Attachment is assessed with the Experiences in Close Relationships (ECR) questionnaire [63,64] and the Dutch Attachment Style Questionnaire [65]. Mentalizing is measured using the Reflective Functioning Questionnaire (RFQ).

The ECR questionnaire [63,64] measures adult attachment in romantic relationships. It contains two subscales, Anxiety about rejection and abandonment (e.g., “I worry about being abandoned”) and Avoidance of intimacy (“I feel very uncomfortable when my partner wants to have a close connection to me”), both consisting of 18 items. Individuals are asked to rate each statement on a seven-point Likert scale ranging from 1 (disagree strongly) to 7 (agree strongly).

The Dutch Attachment Style Questionnaire [65] is a measure of attachment to others in general (and thus not to a specific person as is the case for the ECR). The questionnaire consists of 24 items and measures the following four attachment styles defined by Bartholomew and Horowitz [66]: (1) secure attachment, consisting of eight items (e.g., “I trust others to be there when I need them”), (2) avoidant attachment, consisting of five items (e.g., “It is important for me to be independent”), (3) preoccupied attachment, consisting of seven items (e.g., “I have the feeling that most of the time I like others better than they like me”) and (4) anxious attachment style, consisting of four items (e.g., “I am afraid that others will betray me when I become too close to them”). All items are scored on a five-point Likert scale ranging from 1 (strongly disagree) to 5 (strongly agree). The internal consistency ranges from .61 (Avoidant) to .86 (Preoccupied) [65].

The RFQ [67] consists of 57 items with ratings on a seven-point scale (1 = Strongly disagree, 7 = Strongly agree). An example of a statement is “It costs me a lot of time to understand the thoughts and feelings of other people”. The internal consistency of the RFQ is good (α = .77) and the test-retest reliability is high (α = .78) [67].

##### Quality of life

Quality of life is measured using the EuroQol EQ-5D-3 L [68]. This self-report questionnaire provides a simple method to capture health problems according to a five-dimensional classification: mobility, self-care, usual activities, pain/discomfort and anxiety/depression. Each dimension is divided into three levels: no problem, moderate problems, extreme problems. The five dimensions can be summarized into a “value”, based on the preferences of the general public. These values can be used as societal weights for the calculation of quality-adjusted life years (QALYs) in health economic evaluations (see also below). To calculate these societal weights, we will use a Dutch validation study [69,70]. Next to the five dimensions, the EuroQol presents a vertical visual analogue scale, ranging from 0 (worst imaginable health) to 100 (best imaginable health). The values are seen as representing patients’ values, in contrary to the societal weight based on the five dimensions. The reliability of the EQ-5D-3 L has been investigated and found to be acceptable [71] and it has shown to be sensitive to change in patients with personality disorders [4,72].

##### Mental-health-related functional impairment

The Sheehan Disability Scale (SDS) [73] was developed to assess functional impairment in three inter-related domains: work/school, social and family life. It is a brief self-report tool consisting of four questions. The patient rates the extent to which work/school, social life and home life or family responsibilities are impaired by his or her symptoms on a 10-point visual analogue scale. An example question is: “I do not function well at work because of my problems”. The internal consistency of the SDS total score is good (Cronbach’s α = .89) and test-retest reliability for the SDS total score is acceptable (ICC = .73) [74].

##### DSM-IV Axis-I and Axis-II-diagnoses

The SCID-I [40,75] is used to diagnose Axis I disorders at intake and at 36 months follow-up. The SCID-II [38,76] is used for diagnosing Axis II personality disorders at intake and at 36 months follow-up. The interviewer is an MSc-level psychologist, who was trained by an expert trainer in the SCID-II. No interrater reliability data were collected in this study. Previous research has shown, however, that the SCID-II has good interrater reliability and test-retest interrater reliability in adults [77,78].

##### Patient adherence to the treatment

Patients’ adherence to the treatment is derived from the realized dose. The clinical staff record the type, number, and duration of all attended sessions and the number of missed sessions and reason for absence. Based on this information, we will calculate the proportion of missed sessions as a quantitative index of treatment adherence.

##### Costs

The intervention costs of MBT-DH and TAU are calculated using a mixture of top-down and bottom-up approaches. The intervention costs estimates will include personnel costs, implementation costs (e.g., hosting and coaching) and any other overhead costs associated with the treatment. Medical costs beyond the intervention costs of MBT-DH and TAU are calculated using the Trimbos and Institute for Medical Technology Assessment (iMTA) Questionnaire on Costs Associated with Psychiatric Illness (TiC-P: [79]). The TiC-P will be used to measure health care utilization at baseline and after 6, 12, 18, 24 and 36 months.

The first part of the TiC-P consists of questions on (1) the number of visits to, e.g., a general practitioner, psychiatrist (outside TAU or MBT-DH), medical specialist, physiotherapist or alternative health practitioner; (2) the day care/hospital lengths of stay (outside TAU or MBT-DH); and (3) the use of medication in the 4 weeks prior to filling out the questionnaire. These values are multiplied with unit prices of the corresponding health care services according to the Dutch manual for costing studies in health care [80]. The unit prices for 2010 will be adjusted to 2014 prices using the Consumer Price Index [81]. As the mean direct costs are measured per 4 weeks, we will multiply these values by 13 to estimate the annual costs.

The TiC-P also asks the patient to report any productivity losses, that is, absence from work or reduced productivity at work. This report is used to estimate the so-called “friction costs”: the monetary representation of the replacement of the labour. The friction-cost method takes the employer's perspective, and counts as lost only those hours not worked until another employee takes over the patient's work. This is a more conservative estimate than the so-called “human capital method”, which relates productivity costs one-to-one to the labour costs of the patient. The choice between friction costs and human capital is still a subject of debate among economists. In this study, we chose the more conservative friction-cost method [82].

### Questionnaires completed by therapists

Therapists complete questionnaires at 6, 12, 18, 24, 30 and 36 months after the start of treatment of each patient regarding treatment alliance, functional impairment of the patient, the Clinical Global Impression scale (CGI) and medication adherence.

#### Treatment alliance

The Helping Alliance Questionnaire (HAQ) is based on two types of “helping alliance”. Type I is about the experience of the therapist supporting and helping the patient. Type II is about the experience of joint collaboration [83]. Luborsky and colleagues expanded the original HAQ from 11 to 19 questions [84]. There is both a patient and a therapist version, but in this study only the therapist version is used. Items are rated on a six-point Likert scale (1 = I strongly feel it is not true; 6 = I strongly feel it is true). An example question is “My patient believes I’m experienced”. The total score is the mean of the item scores. The HAQ-II shows good internal consistency (α ranging from .90 to .93) [85] and test-retest reliability (.56) [84].

#### Functional impairment of the patient

We adapted the SDS [73] so that therapists can rate the functional impairment of the patient in three domains: work/school, social and family life.

#### Clinical global impression

The CGI [86] provides a summary of an individual’s clinical functioning as assessed by the therapist. The CGI consists of two ratings: Severity of Illness (CGI-S) at the moment of contact, and Global Improvement (CGI-I) of the patient since the start of treatment. Both use a seven-step categorical scale: for the CGI-S, ranging from 0 (normal, not at all ill) to 7 (among the most extremely ill patients), and for the CGI-I, from 0 (very much improved) to 7 (very much worse).

### Sample size and power calculation

Based on records of 2008, we estimated the number of new referrals per year to Arkin and De Viersprong. On average, the total annual number of new referrals was 265 at De Viersprong and 230 at Arkin. Out of all newly referred patients, 154 suffered from a severe BPD and met the inclusion criteria for this study. Assuming a refusal rate of 35%, this means that on average 8.1 patients could be included each month. Therefore, we expected to be able to recruit and assess 108 patients (54 in each arm) during a recruitment window of 14 months. Figure 1 shows the expected flow of participants from recruitment through the beginning of the study.

In this study the primary clinical outcome variable is the total score on the BPDSI, an index of the severity of BPD manifestations. Bales and colleagues [35] found a large effect size of 1.23 on the BPDSI after 18 months of MBT-DH, in a sample of 45 BPD patients. Although Bateman and Fonagy [33] used another index for the severity of borderline symptoms (the Zanarini Rating Scale for Borderline Personality Disorder, a clinician-administered scale for the assessment of change in DSM-IV borderline psychopathology [87]), they also found a large effect size 5 years after MBT (a difference of 64% between TAU [mean = 15.1, SD = 5.3) and MBT-DH [mean = 5.5, SD = 5.2; effect size = 1.8]). Although within-group effect sizes for MBT can be expected to be large, the specialist TAU investigated in the present study may be more effective than TAU provided in the UK. Yet, given differences in effect sizes in previous trials, particularly in the long term, we expect MBT-DH patients to improve at least 20% more than TAU patients on the BPDSI at 18 months after the start of the intervention. With a sample of 54 patients per group, an effect size of at least .65 on the BPDSI could be detected with a power of 92% (α = .05, *β =* .083), which reflects a reasonable, realistic and clinically relevant effect.

### Statistical analyses

First, to investigate potential differences between the two groups at baseline, we will use parametric and nonparametric descriptive statistics, as appropriate. For the main analyses concentrating on primary and secondary outcomes, linear growth curve models for normally distributed outcome measurements will be used, logistic regression for binary data, and Poisson regression models for ordinal data. Results will be expressed in terms of comparison of the slopes for interval data, odds ratios for binary data and incidence rate ratios for ordinal data. Furthermore, we will perform explorative analyses regarding mechanisms of change using both variable and person-centered approaches. All analyses will be conducted according to intention-to-treat principles.

#### Health economic evaluation

We will estimate the difference in total costs for MBT-DH compared to TAU and the difference in clinically relevant effects of the treatments. By dividing the difference in costs by the difference in effectiveness, the incremental cost-effectiveness ratio (ICER) will be estimated. The ICER represents the extra amount of money that has to be invested or will be saved to gain or lose on extra unit of effect.

In the economic evaluation, all relevant costs and effects will be taken into account, meaning that we will use a so-called societal perspective, which is preferred in economic evaluations in The Netherlands [88]. The costs will include all costs made, that is, intervention costs, direct and indirect medical costs, as well as productivity losses and costs made elsewhere in the health care system. Using the societal perspective, clinically relevant effects are those effects that are meaningful for society. Moreover, in order to compare effects between allocations in health care, effects should be expressed in generic terms. We will therefore use QALYs and will express the cost-effectiveness and its ratio as cost per QALY. The use of QALYs is advised in guidelines for cost-effectiveness analysis, especially when main effects are expected in quality of life [89].

Our primary cost-effectiveness ratio will be estimated using empirical data only and therefore using a 3-year time horizon, which is equal to the trial duration. Cost data are generally highly skewed, and QALY scores and ICER values are not distributed normally in most cases. Therefore, the uncertainty intervals around the mean costs, mean effects and mean ICER values will be estimated using bootstrap simulations with 1000 replications [90-92]. These results will be graphically presented in cost-effectiveness planes. Various societal willingness-to-pay values will be used to estimate net monetary benefits. These net monetary benefits are then used to derive cost-effectiveness acceptability curves.

We will also explore the long-term costs and effects of MBT-DH and TAU from a societal perspective. One way of achieving this would be to use a Markov model to explore the long-term cost-effectiveness of the intervention under study. However, health stages in a Markov model are often predefined, discrete stages, where they should reflect the biological or theoretical understanding of the condition being modelled [93,94]. Because in this trial the BPDSI, which is a continuous measure, is the primary outcome measure, it does not allow for an unambiguous definition of health states to model disease progression. Having heterogeneous health states may be controversial, as it means that a wide range of patients may be clustered in one health state. In order to explore the cost-effectiveness using a Markov model in our study, we will therefore define health states on the basis of a cut-off score on one of the outcome measures such as the BPDSI or the OQ-45. We will extend the cost-effectiveness model results after the duration of the trial using different assumptions based on, for example, the literature. Sensitivity analysis will be performed in order to investigate the influence of those assumptions on the model results.

## Discussion

Although MBT-DH is a promising psychodynamic treatment for BPD [1,24-28], so far only two RCTs have compared MBT-DH to specialist TAU [31,34]. Given the relative paucity of data supporting the efficacy and cost-effectiveness of MBT-DH, the possible influence of researcher allegiance in one of the trials [31], potential spill-over effects in another trial [34], and problems with the generalization of findings to mental health systems in other countries, more research is urgently needed. Further, the only health economic study of MBT-DH did not use state-of-the-art cost-effectiveness methodologies [32]. To assess the efficacy and cost-effectiveness of MBT-DH in The Netherlands, the current study aims to compare MBT-DH with specialist TAU using state-of-the-art cost-effectiveness analyses. Further, because the current study is a multi-site trial, and treatments in both treatment arms are conducted by different therapists, the risk of spill-over effects is minimal.

We expect both MBT-DH and TAU to lead to significant reductions in primary and secondary outcome measures, including symptomatic distress and interpersonal functioning. However, we expect MBT-DH to outperform TAU, particularly at 36 months follow-up.

To date, no studies have examined the purported mechanism of change in MBT in the context of a randomized trial and there is a pressing need to provide evidence for presumed mechanisms of change in current evidence-based treatments [37]. Aside from providing new data on the efficacy and cost-effectiveness of MBT-DH, this trial also promises to provide a better understanding of the mechanisms of change in MBT.

## Abbreviations

BDI: Beck depression inventory; BPD: Borderline personality disorder; BPDSI: Borderline personality disorder severity index; BSI: Brief symptom inventory; CGI: Clinical global impression scale; DAPP-SF: Dimensional assessment of personality pathology – short form; ECR: Experiences in close relationships; GAF: Global assessment of functioning; GSI: Global severity index; HAQ: Helping alliance questionnaire; ICER: Incremental cost-effectiveness ratio; IIP-64: Inventory of interpersonal problems; MATE: Measurements in the addictions for triage and evaluation; MBT-DH: Day hospital mentalization-based treatment; MBT: Mentalization-based treatment; MBT-IOP: Intensive outpatient mentalization-based treatment; OQ-45: Outcome questionnaire; PAI-BOR: Personality assessment inventory-borderline; QALY: Quality-adjusted life year; RCT: Randomized controlled trial; RFQ: Reflective functioning questionnaire; SCID-I: Structured clinical interview for DSM-IV Axis I personality disorders; SCID-II: Structured clinical interview for DSM-IV Axis II personality disorders; SDS: Sheehan disability scale; SIPP-SF: Severity indices of personality problems – short form; SSHI: Suicide and self-harm inventory; STEPPS: Systems training for emotional predictability and problem-solving; TAU: Treatment as usual; TiC-P: Trimbos and Institute for Medical Technology Assessment Questionnaire on Costs Associated with Psychiatric Illness.

## Competing interests

The authors declare that they have no competing interests.

## Authors’ contributions

EMPL drafted the first version of the manuscript and maintained the lead in the writing process. DW was responsible for the coordination of the study and collecting the data. JJMD leads the research project. JJMD, MJK and JP developed the study design and wrote the study proposal. JP developed the statistical design and randomization procedure, manages the data flow and will perform the statistical analyses together with EMPL. HVE is responsible for the cost-effectiveness analyses. AJVB was coordinator at the MBT-unit during the study. PL, JJVB, HVE and MN made substantial contributions to the revision of the manuscript. All authors provided comments, read and approved the final manuscript.

## Pre-publication history

The pre-publication history for this paper can be accessed here:

http://www.biomedcentral.com/1471-244X/14/149/prepub
